# Non-communicable diseases and reproductive health in sub-Saharan Africa: bridging the policy-implementation gaps

**DOI:** 10.1186/s12978-020-0857-8

**Published:** 2020-01-23

**Authors:** Sanni Yaya, K. Srikanth Reddy, José M. Belizán, Verónica Pingray

**Affiliations:** 1 School of International Development and Global Studies, Faculty of Social Sciences, University of Ottawa, Ottawa, Canada; 2 The George Institute for Global Health, The University of Oxford, Oxford, UK; 3 Bruyere Research Institute, University of Ottawa, Ottawa, Canada; 4 Department of Mother and Child Health Research, Institute for Clinical Effectiveness and Health Policy (IECS-CONICET), Buenos Aires, Argentina

Sub-Saharan Africa (SSA) region is a home for over one billion population distributed in 46 different countries. Over the decades, this region has confronted with high disease burden accounting around 24% of the global disease burden [1]. Traditionally, communicable diseases such as HIV, TB, and Malaria have long been the most prominent contributors to the disease burden. However, in the last two decades, the region has witnessed an epidemiological transition to non-communicable diseases (NCDs) [2]. Around 80% of all NCDs deaths occur in low and middle-income countries. It is projected that by 2020, NCDs will account for 27% of mortality in SSA [3]. Majority of NCDs related deaths can be prevented by addressing the common modifiable risk factors for NCDs include tobacco use, physical inactivity, the harmful use of alcohol, and unhealthy diet [4].

The NCDs and reproductive health morbidity and mortality, has become a significant part of the disease burden in the region requiring a rethink on policy prioritisation and implementation to minimise the burden.

The WHO defines reproductive health as a “state of complete physical, mental and social well-being and not merely the absence of disease or infirmity, in all matters relating to the reproductive system and its functions and processes.” This implies that people are able to have a satisfying and safe sex life and that they can reproduce and the freedom to decide if, when, and how often to do so [5]. However, reproductive health among women was often constrained by socio-cultural and economic factors across the societies, particularly in SSA. The access to abortion and contraception are severely restricted, contributing to high maternal mortalities and poor sexual and reproductive health outcomes [6–9]. Reproductive and maternal morbidities increase the risk of maternal mortality, stillbirth, neonatal death. Women facing pregnancy related complications are predisposed to immediate and long-term disabilities and infertility as well as adverse psychological and socio-economic consequences. SSA region faces high burden of maternal mortality, stillbirth and neonatal mortality rates [10]. In 2015, two-thirds of all maternal deaths worldwide occurred in SSA (546 maternal deaths per 100,000 live births [11]. SSA women in reproductive age (15–49 years) have low contraceptive prevalence rate (28%), and about half of them do not receive four or more antenatal check-ups (54%), and do not give birth in health facilities (52%). Further, adolescent girls and young women in the region face high risk of unintended pregnancies, sexually transmitted infections, HIV, and exposure to violence [12]. Further, 244,000 infants in the region annually become infected with HIV during pregnancy and delivery (115,000) or breast-feeding (129,000) [13].

The unmet needs of sexual and reproductive health services among SSA women are coupled with the rising burden of non-communicable diseases. NCDs account for almost 65% of women’s deaths globally, and the majority of these deaths occur in LMICs and are premature. In recent years, NCDs among women of reproductive age has doubled in many African countries [14]. Also, there are established linkages between NCDs and reproductive health. Several NCDs risk factors adversely affect the reproductive health of women. For example, obesity, CVDs, hypertension, hyperglycemia, and gestational diabetes predispose pregnant women at higher risk of menstrual problems, hypertension in pregnancy, caesarean sections, post-natal complications and maternal mortality [10]. Obesity also increases the odds of developing CVDs and cancers in women [15, 16]. In a recent study, the prevalence of obesity across 32 Sub-Saharan African countries ranged from 1.1% in Madagascar to 23.0% in Swaziland [17]. A study shows that that women at the beginning of pregnancy 73.7% and 60.2% women in South Africa and Zimbabwe, respectively started their pregnancy with BMI above normal (BMI ≥ 25) [18].

Epidemiological, clinical and animal studies have shown the modelling effect of fetal life on NCDs diseases of adult life, like hypertension, coronary heart disease and diabetes [19]. Furthermore, nutrients restriction during fetal life have shown higher blood pressure in the progeny. Animal studies have shown that protein restriction and calcium restriction during pregnancy involved hypertension of the progeny [20]. In humans, calcium supplementation during pregnancy involved a reduction of hypertension of children whose mothers were supplemented in comparison with a placebo group [21]. In a previous mentioned study done in South Africa and Zimbabwe the prevalence of inadequate micronutrient intake from food sources was high in both countries. For the most basic micronutrients like iron, calcium, folate and zinc, the percentage of women below requirements was above 90% in both countries [18].

Further, research suggests that obesity is linked to polycystic ovary syndrome (PCOS) in women. Moreover, obese women with PCOS have worse metabolic and reproductive outcomes [22]. The hypertensive disorders of pregnancy is one of the major cause of maternal deaths and stillbirths in the region [10].

African Union (AU) is at the forefront of shaping the national health priorities including women and child health. The AU, despite being the first to develop African Charter of Human and People’s Rights on the Rights of Women in Africa that mandates state provision of comprehensive reproductive and sexual health services, is yet to be a reality and achieve the objective [23]. Building on Millennium Development Goal’s (MDGs) progress on reducing maternal and child mortality and malnutrition, combating infectious diseases, an updated Global Strategy for Women’s and Children’s Health (2016–2030) was launched in 2016 [24, 25]. This universal and equity-based strategy aims to end preventable deaths among women by reducing global maternal mortality to less than 70 per 100,000 live births and reduce by one-third premature mortality from NCDs and promote mental health and well-being. The reproductive health policies must be in alignment with this global strategy to minimize the mortalities due to NCDs risk factors linked to reproductive health. And also, to optimize better sexual and reproductive health in the SSA.

At the individual level, prevention and continuum of care approach through “life-course” are essential for the prevention of NCDs. While most NCD related health outcomes cause morbidity and mortality during adulthood, exposure to risk factors begins early in life, and these behaviours often get established for the lifetime. WHO recommends that NCD prevention and control measures to consider health and social needs and reduce exposure to risk factors at all stages of the life course. Similarly, improving reproductive health outcomes calls for a comprehensive understanding of women’s health throughout the life course. The global strategy for women’s health adopts an integrated life course approach and underscores the need for investment in child and adolescent health and development [24].

At the systemic level, a comprehensive approach requires multipronged strategies focussing on prevention within primary care through community-based programs, health promotion, and cost-effective policies that target the whole population as well as high-risk individuals. Both NCDs and reproductive health thus needs to be addressed through both “population-based” and “high-risk individual” based approaches to empower individuals and populations to make healthier choices and access to health services. The global strategy recommends integrating NCDs prevention and treatment with women’s, children’s, and adolescents’ health care [24, 25]. The population-level approaches include increasing awareness, creating a conducive environment and instituting public health policies while high-risk individual-level approaches include early diagnosis and management and treatment of disease and need to integrate into the health systems in SSA.

At the policy level, various bodies have emphasised the need for a collective response towards addressing health issues as it cuts across the sectors. The WHO has emphasized that effective NCD prevention and control require “health-in-all policies and whole-of-government approaches” across sectors, involving a range of ministries outside health such as health, agriculture, communication, education, employment, social welfare, social and economic development, sports, trade and industry transport, urban planning and others. Similarly, Global Strategy for Women’s and Children’s Health also emphasise the adoption of a multi-sectoral approach to develop and monitor interventions outside the health sector and asserts the countries to build governance mechanisms and capacity to facilitate multisector actor and cross-sector collaborations [25].

## Conclusion

Most risk behaviours for NCDs and reproductive health are modifiable, and the related morbidity and mortality is preventable. Yet, the progress is largely determined by socioeconomic, demographic, political factors. Strengthening national policies, health systems, country-level surveillance, and monitoring systems, and creating sustainable partnerships and advocacy are key strategies towards addressing these health issues. However, implementation gaps remain contributing not only NCDs mortality, but also reproductive health. Identification of common risk factors and linkages among the NCDs and reproductive, would help intersectoral action and integration of health care services for the prevention of mortalities in respective sub-Saharan African counties. Such an effort, while contributing to better health outcome in region, also help accelerating the Sustainable Development Goals and targets, particularly SDG 3.4, 3.6, 3.7 and SDG11.2. Therefore, the journal encourages global public health scholars, particularly from sub-Saharan African countries to reflect upon these considerations and submission of manuscripts.
